# Identification and Expression Patterns of Opsin Genes in a Forest Insect, *Dendrolimus punctatus*

**DOI:** 10.3390/insects11020116

**Published:** 2020-02-11

**Authors:** Sufang Zhang, Xiangbo Kong, Fu Liu, Zhen Zhang

**Affiliations:** Key Laboratory of Forest Protection of National Forestry and Grassland Administration, Research Institute of Forest Ecology, Environment and Protection, Chinese Academy of Forestry, Beijing 100091, China; zhangsf@caf.ac.cn (S.Z.); xbkong@sina.com (X.K.); liufu2006@163.com (F.L.)

**Keywords:** vision, long wave sensitive opsin, adult, expression level

## Abstract

*Dendrolimus punctatus* walker (Lepidoptera: Lasiocampidae) is the most serious coniferous forest defoliator in China. This species has long life history, and shows different activity rhythms and light response behaviors at larval and adult stages. Insect vision system play important roles for survival and reproduction, and disturbance of photoreception may help us to control this pest. However, we know little about the visual system of *D. punctatus*. As opsins are the most important genes determining photoreceptor sensitivity of insects, we identified opsins of *D. punctatus* and analyzed their expression patterns at different development stages in this study. Four opsin genes were identified based on our transcriptome data. Phylogenetic analysis showed that there are three classical ultraviolet (UV), blue, and long-wavelength (LW) light sensitive opsin genes, and another UV-like opsin as homolog of a circadian photoreceptor, Rh7, in Drosophila melanogaster and other insects. Expression analysis indicated that the UV and UV-like opsins expression levels only fluctuated slightly during whole life stages of *D. punctatus*, while Blue and LW opsins were up-regulated many times at adult stage. Interestingly, the ratio of UV-opsin was much higher in eggs and larvae stages, and lower in pupa and adult stages; reversely, LW-opsin showed extremely high relative ratio in pupa and adult stages. High expression level of LW opsin in the adult stage may correlate to the nocturnal lifestyles of this species at adult stage, and different ratios of UV and LW opsins in larval and adult stages may help to explain the different visual ecologies of these two development stages of *D. punctatus*. This work is the foundation for further research of opsin functions and vision mechanisms of *D. punctatus*.

## 1. Introduction

The interactions between pests and host plants rely on several sensory systems, such as olfaction, gustation, audition, and vision. Among these sensory systems, vision of insects plays important roles during foraging [1], host identification [2], mate choice [3], and predatory defense [4]. Some of these behaviors may be guided by a combination of sensory systems [5]. However, most researches about insect sensory systems were focused on olfaction [6] or gustation [7], and there have been much less focused on vision [8]. With the development of research techniques, the functions of vision in insect survival have attracted more attention recently [9].

Vision is underpinned by visual pigment molecules that contain a seven transmembrane opsin and a chromophore, usually 11-cis retinal. The amino acid sequence of the opsins and chromophores determine the spectral sensitivity of photoreceptors together [10,11,12]. Generally, opsins of insects can be divided into three types based on their pigment absorption spectral peaks, which are ultraviolet-sensitive (UV opsin, 325–400 nm), blue light-sensitive (Blue opsin, 400–500 nm), and long-wavelength-sensitive proteins (LW opsin, >500 nm) [13]. The UV, Blue, and LW opsins are three classic genes in insect visual system, and many insects such as hymenopterans contain all the three opsins, but less or more than three opsins appear in other insects due to gene duplication or losses [14,15]. For example, *Apis mellifera* contain three opsin genes corresponding to UV, Blue, and LW spectral peaks [16], six opsins were found in *Drosophila* [11], and six opsins were identified in *Papilio glaucus* [17]. The expression of opsin was also species specific, based on the evolution of insect visual systems shaped by the light environment [18,19,20].

In lepidopteran insects, the photoreception and compound eyes are highly developed, and adults of two main insects types in this order, butterflies and moths, have different responses to light rhythms. The butterflies are diurnal insects and some researchers have illustrated their adaption mechanisms to lights [21,22,23]. However, only very little work was related to the vision mechanisms of moths, which are most typical nocturnal insects [18,19]. Interestingly, the light sensitivity of moths is much higher than butterflies, because the moths have excellent low-light vision [24,25]. Moths are very sensitive to light traps, which are usually used in insect surveys and mass trapping of adults in pest management programs [26]. Determining the opsin copies and patterns of expressions in nocturnal moths may lead to more accurate regulation of their phototactic behaviors and assist in their management [27,28,29,30].

*Dendrolimus punctatus* walker (Lepidoptera: Lasiocampidae) is the most serious coniferous forest defoliator in south China, and this pest outbreak periodically [31]. Once outbreak, *D. punctatus* population density achieves a very high level, and the larvae feed on pine needles intensively, which may reduce the cone yield, affect tree growth, and even cause tree death [32]. Outbreaks of *D. punctatus* stimulated extensive insecticide application, and effected on biodiversity and natural enemies in the ecosystem. Thus, it is urgent to develop new methods to control this forest pest. The sensory systems connect insects with various information in the environment, and sensory block may be an efficient method to control *D. punctatus*. Until now, the olfactory sensory mechanisms of *D. punctatus* have been explored extensively [33,34,35]. However, little is known about another important sensory system, vision, of this pest. *D. punctatus* occurs one to four generations per year depending on the climate of occurring places. The eggs or adult stages maintain approximately one week, the pupae maintain approximately two weeks, and the larvae stage can fluctuate between one month and six months depending on different generations in a year [31]. It is worth mentioning that *D. punctatus* adults and larvae have different visual ecologies, because adults are nocturnal, and larvae are also active at daytime. However, the mechanisms that control their visual ecologies are unclear. As opsins are genes that underpin light response in insects, it is essential to identify and explore the expression patterns of opsins in this pest, and this may be the foundation for further research of vision mechanisms.

## 2. Materials and Methods

### 2.1. Identification of Opsins

The transcriptome data of *D. punctatus* at four development stages (including egg, larva, pupa, and adult) were same with our previous study [35], we also referenced the SRA database downloaded transcriptome data (accession SRP064724). To identify the opsins of *D. punctatus*, the contigs were analyzed with tBLASTx at NCBI, and the contigs hit with opsins of other species were further identified with their open reading frames (ORFs) by blast searches (http://blast.ncbi.nlm.nih.gov/Blast.cgi). The identified opsins of *D. punctatus* was submitted to NCBI with accession number MN868672-MN868675.

### 2.2. Sequencing Analysis and Phylogenetic Tree Construction

*D. punctatus* opsins were aligned with DNAMAN (Version 8, LynnonBiosoft, QC, Canada). With TMHMM Server (Version 2.0, http://www.cbs.dtu.dk/services/TMHMM/), the transmembrane domains were identified. Protein sequences of opsins from *D. punctatus* and other insects were used to perform phylogenetic analysis. Both neighbor-joining (NJ) and maximum likelihood (ML) trees were constructed to confirm the results. The ML tree was constructed by MEGAX with 500 bootstrap replications, and Le and Gascuel, 2008 model [36] with frequencies and Gamma distributed sites (LG+F+G) model of evolution was selected based on result of MEGA’s model test. NJ tree was constructed using MEGAX with 10000 bootstrap replications and Poisson model + uniform rates. The tree was annotated in Adobe illustrator (Adobe Systems).

### 2.3. Quantification of Gene Expression Levels with Real-time PCR

We collected the pupae of *D. punctatus* in Quanzhou County, Guilin city, Guangxi Province, China, in May of 2018. The pupae were reared at 50 ± 10% relative humidity and 26 ± 2 °C with a photoperiod of 16 h light: 8 h dark. After emergence, some adults were immediately frozen in liquid nitrogen, and some of the adults were continuously reared for samples collection of various developmental stages. Eggs (approximately 2–3 d), larvae (a mix of all larval instars), and pupae (approximately 5 d) samples were collected and frozen in liquid nitrogen. Samples of each developmental stage were collected from more than five insects in the morning of a day; and three biological replications for each development stage were prepared.

Then we extracted the total RNA from whole tissue of each sample with TRIzol, according to the manufacturer’s instructions (Invitrogen, Carlsbad, CA, USA), and monitored the RNA degradation and contamination on 1.2% agarose gels. With 1μg of total RNA prepared above, cDNA templates were synthesized with M-MLV reverse transcriptase (Promega, USA), according to the manufacturer’s instructions.

Primers that resulting 100–250 bp products were designed from the unigene sequences (Table 1). Normal PCR using qTaq DNA polymerase (TaKaRa, Dalian, Liaoning, China) and sequencing were performed to verify the correct products and no primer dimers. We used 2^−^^ΔΔ^CT method to measure the relative expression levels of opsin genes. As our pervious researches indicated that *beta-actin* is a reliable reference gene for real-time PCR in *Dendrolimus* insects [35], the expression level of opsin genes was normalized by *beta-actin*. The Real-time PCR were performed in 20 μL reaction volume with 10μL of 2× SuperReal PreMix (Tiangen, Beijing, China) on an ABI7500 (USA), and the program was set as follow: 2 min at 95 °C, then 40 cycles of 20 s at 95 °C, 20 s at 58 °C, and 20 s at 72 °C, at last 58 °C to 95 °C to perform melting curve analysis and evaluate the specificity of the real-time PCR products. Triplicates (technical replicates) were performed for each reaction, and three independent biological replicates were performed for each development stage.

Gene expression level comparisons were performed by the SPSS statistical program (version 18.0; SPSS Inc., USA) with Analysis of variance (ANOVA) and Turkey’s honestly significant difference (HSD) test.

## 3. Results

### 3.1. Opsin Identification and Sequence Analysis

**Figure 1:**
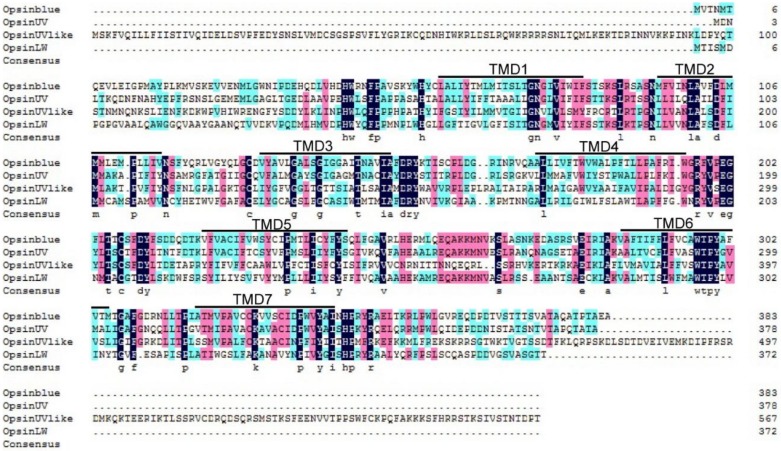
Sequence alignment of four opsins from *Dendrolimus punctatus*. The seven transmembrane domains are marked above the alignments as TMD1–7.

Based on the transcriptome data, we identified four opsin genes in *D. punctatus*, named as *DpunOpsin blue*, *DpunOpsin UV*, *DpunOpsin UV-like*, and *DpunOpsin LW*, respectively. These four opsins encoded 383, 378, 568, and 372 amino acid residue proteins, respectively. *DpunOpsin UV like* gene have a longer 5′ and 3′ end than other opsin genes, as shown in the aligned results, and all the four proteins have seven transmembrane domains (Figure 1).

### 3.2. Phylogenetic Analysis of the Opsins

**Figure 2:**
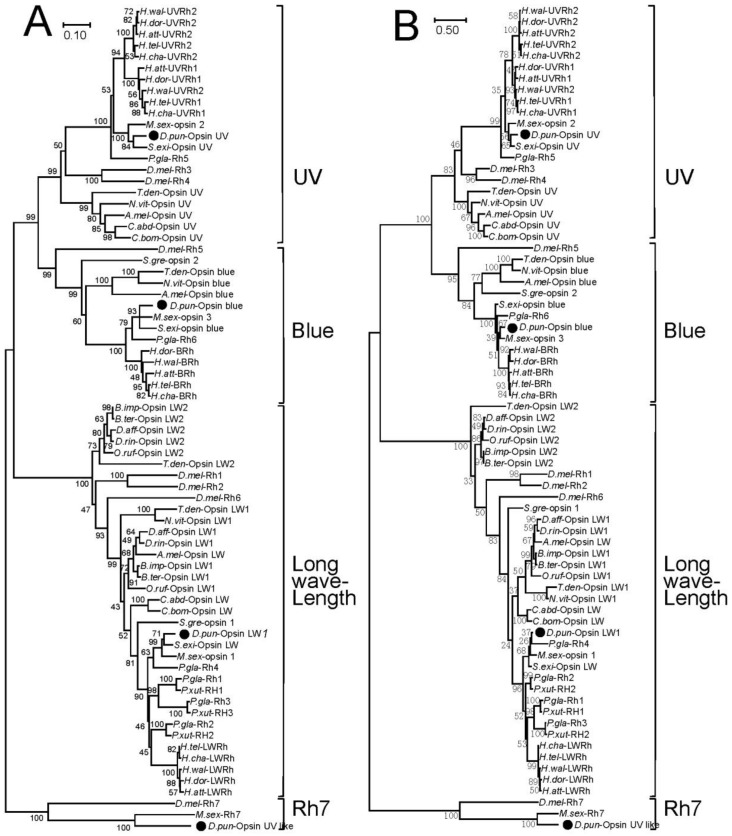
Neighbor-joining (NJ) and maximum likelihood (ML) trees based on protein sequences of opsin from *Dendrolimus punctatus* and other insects. Opsins from *Dendrolimus punctatus* (*D.pun*); *Drosophila melanogaster* (*D.mel*); *Apis mellifera* (*A.mel*); *Trichogramma dendrolimi* (*T.den*); *Camponotus abdominalis* (*C.abd*); *Cataglyphis bombycinus* (*C.bom*); *Manduca sexta* (*M.sex*); *Papilio glaucus* (*P.gla*); *P. xuthus* (*P.xut*); *Schistocerca gregaria* (*S.gre*); *Bombus impatiens* (*B.imp*), *B. terrestris* (*B.ter*); *Diadasia afflicta* (*D.aff*); *D. rinconis* (*D.rin*); *Osmia rufa* (*O.ruf*); *Heliconius wallacei* (*H.wal*); *Heliconius telesiphe* (*H.tel*); *Heliconius doris* (*H.dor*); *Heliconius charithonia* (*H.cha*); *Heliconius atthis* (*H.att*); *Spodoptera exigua* (*S.exi*) (**A**) NJ tree constructed using MEGAX with 10000 bootstrap replications and Poisson model+uniform rates. (**B**) ML tree constructed by MEGAX with 500 bootstrap replications, and Le and Gascuel, 2008 model with frequencies and Gamma distributed sites (LG+F+G) model of evolution. The bar indicates phylogenetic distance, and the black points refer to the opsins of *Dendrolimus punctatus*.

Two phylogenetic comparisons with opsin protein sequences from *D. punctatus* and other species, including model insects such as *Drosophila melanogaster*, *Apis mellifera*, were constructed (Figure 2). Both NJ (Figure 2A) and ML (Figure 2B) trees showed that UV, Blue, and LW opsins from *D. punctatus* were clustered into three branches that similar to other insects, the UV like opsin gene of *D. punctatus* were separated to a single branch cluster with a circadian photoreceptor, Rh7, in *Drosophila melanogaster* and other insects.

### 3.3. Expression Levels of the Opsins during Four Development Stages

**Figure 3:**
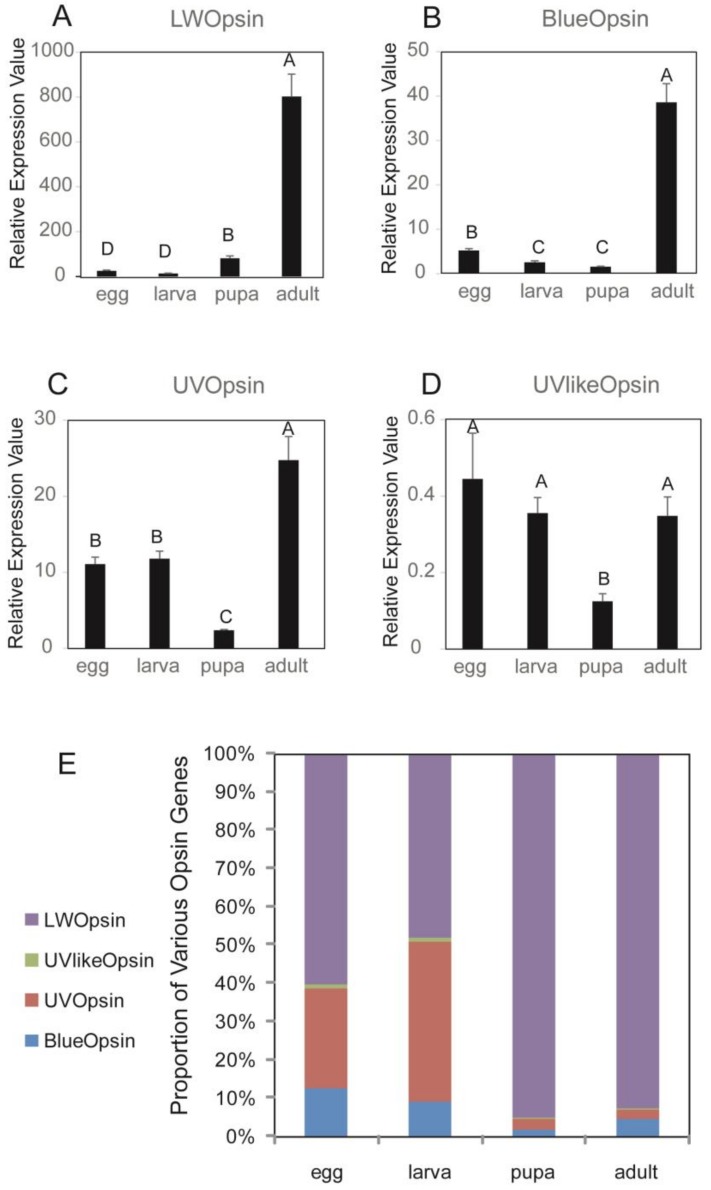
Development stages-dependent expression levels and ratios of the four opsin genes in *D. punctatus*. (**A**) expression levels of *DpunOpsin LW*; (**B**) expression levels of *DpunOpsin blue*; (**C**) expression levels of *DpunOpsin UV*; (**D**) expression levels of *DpunOpsin UV=like*; (**E**) ratios of various opsins at each developmental stage. Real-time PCR was performed using various tissue samples of *Dendrolimus punctatus*, and the expression values were normalized to that of β-actin in A-D. Three replications were performed, and the expression data were shown as mean ± SE in A-D and mean value in E. The letters above each bar of A-D indicate significant differences according to a Tukey’ honestly significant difference (HSD) test (*p* < 0.05).

The expression levels of opsin genes relative to *beta-actin* during four development stages were different (Figure 3). Among the four opsins, *DpunOpsin LW* showed much higher expression level than other three genes, with about 20 times of *DpunOpsin Blue*, 30 times of *DpunOpsin UV* at adult stage. The *DpunOpsin UV-like* gene only showed very low expression level (Figure 3D). During different development stages, *DpunOpsin LW* and *DpunOpsin Blue* both up regulated their expression at adult stages greatly; but *DpunOpsin UV* and *DpunOpsin UV-like* opsin expression levels only fluctuated slightly from egg to adult. To further compare the ratios of various opsins at each developmental stage of *D. punctatus*, we illustrated the proportion of the four opsins gene expression levels at different developmental stages (Figure 3E). The results showed that ratio of *DpunOpsin UV* was much higher in egg and larva stages, and lower in pupa and adult stages; reversely, *DpunOpsin LW* showed extremely high relative ratios in pupa and adult stages. These may help to explain the different visual ecologies of larval and adult stages of *D. punctatus*.

## 4. Discussion

As a serious defoliator of pine forests, vision system disturbance, such as more efficient light trapping, or genetic manipulation of the critical opsin genes of *D. punctatus*, maybe efficient method to control their population and damage. In this work, we identified and analyzed the important vision genes, opsins, in *D. punctatus*. The results are foundation for further gene function studies and pest photoreception disturbance.

Based on previous studies, there are normally three types of opsins in lepidopteran insects, including butterflies and moths [13,19,30,37]. All the three opsin types were identified in *D. punctatus*, which were UV, B, and LW opsins. Additionally, another UV-like opsin was identified. The phylogenetic analysis showed that this UV-like genes was relatively far from the main UV cluster, and it was separated to a single branch cluster with a circadian photoreceptor, Rh7, in *Drosophila melanogaster* and other insects. The expression level of this UV-like gene was very low compared to other opsins, which was according to that of Rh7 in *Drosophila* [38,39].

Expression analysis of the four opsins in *D. punctatus* showed that their ratios were different at four development stages. Ratios of UV-opsin were much higher in egg and larva stages, and lower in pupa and adult stages. Reversely, LW-opsin showed extremely high relative ratios in pupa and adult stages (Figure 3E). These may explain the different visual ecologies of larval and adult stages of *D. punctatus*. UV opsin showed only slight up regulation from larval to adult stages, but the eye size increased significantly relative to body size in adult stages, so we can still expect a larger relative contribution of UV to the visual system in larvae vs adults. LW opsin is the main one expressed in adults, which is according to the results of *Spodoptera exigua* [30] and *Manduca Sexta* [40]. This situation may correlate with the nocturnal lifestyles of adult moths, as high LW expression level may help the insect to adapt the low light environment during the night [24,25].

A recent study in *S. exigua* showed that LW opsin knockdown led to reduced green light phototactic efficiency in adults [30], confirming that the high expression level of LW opsin gene in adults contribute to phototaxy of moths. However, studies on opsin gene functions of moth larvae are rare and differences in opsin expression patterns between different life stages were not previously known. Thus, the molecular bases of different visual ecologies of larva and adult stages were unclear. Our study gives evidence that the expression ratios of different opsins in larva and adult *D. punctatus* are different, but further in vivo function studies are needed to explore the mechanisms of different visual ecologies in different development stages.

## 5. Conclusions

Four opsin genes of *D. punctatus* were identified based on our transcriptome data of different development stages. Phylogenetic analysis showed that there were three classical opsin genes, which were sensitive to UV, Blue and LW light, and another circadian photoreceptor. Gene expression analysis showed that ratio of *DpunOpsin UV* was much higher in egg and larva stages, and lower in pupa and adult stages; reversely, *DpunOpsin LW* showed extremely high relative ratios in pupa and adult stages. The different expression ratios of *DpunOpsin UV* and *DpunOpsin LW* in different development stages of *D. punctatus* may be adapted to maximize light sensitivity according to the visual ecologies of larval and adult stages.

## Figures and Tables

**Table 1:** Primers used for real-time PCR.

Purpose/Primer Name	Sequence (5′—3′)
*DpunOpsinLW*-5′	GCCTGCGGAACTGACTA
*DpunOpsinLW*-3′	CGACAGCCTGAACAATAAA
*DpunOpsinBlue*-5′	GCTGGAGATGCCTTTGC
*DpunOpsinBlue*-3′	TGCTGGGAGGAGGGTAA
*DpunOpsinUV*-5′	TGTTTCTTATTTGTGGCTTCC
*DpunOpsinUV*-3′	TTGTCGTCGGGTTCGTC
*DpunOpsinUV-L*-5′	TAGCGACTTTATCAGCA
*DpunOpsinUV-L*-3′	CCATAGCCAATATCCAA
*Dpunbeta-Actin*-5′	GCGATCTTACCGACTACCTCA
*Dpunbeta-Actin*-3′	TCTGGGCAACGGAACCT
